# Green‐Synthesized Silver Nanoparticles on Mesoporous Silica‐Coated Carbon Nanotubes: Enhanced Dual Antibacterial and Anticancer Efficacy

**DOI:** 10.1155/bca/9914702

**Published:** 2025-12-09

**Authors:** Su-Ji Ryu, Han-Sol You, Hye-Min Kim, Hyo-Jin An, Jong-Suep Baek

**Affiliations:** ^1^ Department of Bio-Health Convergence, Kangwon National University, Chuncheon, 24341, Republic of Korea, kangwon.ac.kr; ^2^ College of Pharmacy and Institute of Integrated Pharmaceutical Sciences, Kyung Hee University, 26 Kyungheedae-ro Dongdaemun-gu, Seoul, 02447, Republic of Korea, khu.ac.kr; ^3^ BeNatureBioLab, Chuncheon, 24206, Republic of Korea

**Keywords:** antibacterial, mesoporous silica, methicillin resistance *Staphylococcus aureus*, multiwalled carbon nanotubes, silver nanoparticle

## Abstract

This study reports the synthesis and evaluation of novel nanohybrids comprising multi‐walled carbon nanotubes (MWCNTs) coated with mesoporous silica CNTs (MSCNTs) to facilitate uniform silver nanoparticles (AgNPs) formation. The extract of *Angelica gigas* Nakai processed by hot‐melt extrusion (HAE) was employed as a green stabilizing and reducing agent for AgNP synthesis. The resulting MSCNT formulations were comprehensively characterized by transmission electron microscopy (TEM), energy‐dispersive spectroscopy (EDS) mapping, and X‐ray diffraction (XRD). We investigated the influence of HAE concentration on AgNPs formation and distribution on MSCNTs and subsequently evaluated their synergistic effects against methicillin‐resistant *Staphylococcus aureus* (MRSA). Antibacterial activity was assessed through minimum inhibitory concentration (MIC) and minimum bactericidal concentration (MBC), alongside biofilm formation inhibition and disruption activities. Our findings revealed that higher concentrations of HAE significantly improved the uniform distribution of AgNPs on MSCNTs, leading to enhanced antimicrobial activity against MRSA. Furthermore, the MSCNT‐HAE formulations effectively inhibited and disrupted MRSA biofilm formation, a key mechanism of antibiotic resistance. Beyond their antibacterial properties, the cytotoxicity of these nanohybrids was evaluated against both normal human keratinocytes (HaCaT) and triple‐negative breast cancer (MDA‐MB‐231) cells. Further investigations into apoptosis, utilizing cell staining and flow cytometry (FACS), were conducted on MDA‐MB‐231 cells. The results demonstrated that the incorporation of HAE significantly enhanced the selective cytotoxic effects against cancer cells, promoting apoptosis. This research highlights the potential of green‐synthesized AgNPs on MSCNTs as a promising dual‐action therapeutic strategy for combating antibiotic‐resistant bacterial infections and cancer.

## 1. Introduction

The indiscriminate use of antibiotics to treat bacterial infections is leading to increasing problems, such as the emergence of multidrug‐resistant bacteria [1]. The overuse of antibiotics can result in the development of multidrug‐resistant (MDR) bacteria, prolonged infection treatment, and an elevated risk of mortality [2]. *Staphylococcus aureus* (*S. aureus*) infection is one of the most prevalent causes of morbidity and mortality worldwide [3]. β‐Lactams can inhibit bacterial growth by stopping the cell wall synthesis process [4]. Vancomycin is one of the antibiotics used to treat methicillin‐resistant *S. aureus* (MRSA) infections. MRSA, which is resistant to β‐lactam antibiotics, has also been found to be resistant to vancomycin [5]. Most bacteria develop resistance to antibiotics through the formation of biofilms [6]. Since MRSA was first reported in the 1960s, it has been recognized as a pathogen of global concern [7]. MRSA causes staph infections that are difficult to treat due to its resistance to several drugs. It is an antibiotic‐resistant *S. aureus*, a highly adaptable bacterium that causes a variety of diseases, from skin infections to serious systemic diseases such as pneumonia, sepsis, bacteremia, and endocarditis [8]. The emergence of antibiotic resistance and the very slow progress in discovering new antibiotics necessitate the development of alternative treatments to address this problem.

Nanotechnology offers a promising avenue for addressing these challenges, with silver nanoparticles (AgNPs) emerging as a compelling alternative to conventional antibiotics [9, 10]. AgNPs possess unique physicochemical properties, including their diminutive size (< 100 nm), high surface area, and excellent dispersion [11], which contribute to their potent antimicrobial activity. A particularly attractive approach for AgNPs synthesis is green synthesis, which utilizes natural extracts (e.g., from bacteria, fungi, yeast, or plants) as ecofriendly reducing and stabilizing agents [12–14]. Plant extracts, in particular, can enhance AgNPs synthesis and antibacterial efficacy, partly due to their increased water solubility [15]. While the precise antibacterial mechanisms of AgNPs are still being elucidated, they are known to exert their effects by releasing silver ions (Ag^+^), which can disrupt bacterial cell membranes [16], penetrate cell walls and cytoplasmic membranes [17], interfere with ribosomal function, inhibit protein synthesis [18], and inactivate DNA replication and ATP production, ultimately promoting the generation of reactive oxygen species (ROS) that lead to cell death [19, 20].

Multi‐walled carbon nanotubes (MWCNTs) are widely recognized for their cost‐effectiveness and commercial applicability compared to single‐walled CNTs (SWCNTs) [21]. Integrating AgNPs with CNTs has been proposed as an effective strategy to enhance the antimicrobial activity of AgNPs by improving their stability and increasing their effective surface area [22]. However, a significant challenge in utilizing CNTs as carriers is their inherent tendency to aggregate due to van der Waals interactions [23]. Surface modification, such as functionalization with carboxyl groups, has been shown to improve MWCNTs dispersibility and facilitate the uniform formation of AgNPs on their surface [24–27]. MWCNTs decorated with AgNPs benefit from increased surface area, reduced intertube resistance, and crystalline nature [28]. Furthermore, plant extracts have been successfully employed as additives to develop nanohybrids with AgNPs formed on CNTs surfaces [29–31], with examples such as *Camellia sinensis* leaf extract serving as a stabilizing and reducing agent for functionalized MWCNTs [32]. Despite these advancements, achieving uniform particle size and distribution of AgNPs on MWCNTs remains critical for consistent antibacterial activity. Mesoporous silica coating offers an effective solution to this challenge by providing a structured template for uniform AgNPs decoration within its pores, as demonstrated by previous work grafting mesoporous silica onto SWCNTs [33].

**Scheme 1:**
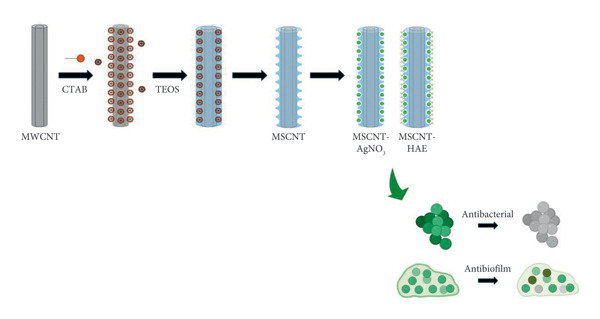
Schematic illustration of the synthesis of MSCNTs containing AgNPs and the evaluation of their antibacterial and antibiofilm activities against MRSA. MWCNT: multiwalled carbon nanotube; CTAB: cetyltrimethylammonium bromide; TEOS: tetraethyl orthosilicate; MSCNT: MWCNT coated with mesoporous silica; AgNO_3_: silver nitrate; AGN: *Angelica gigas* Nakai; MRSA: methicillin‐resistant *Staphylococcus aureus*.

Building upon our previous research on Gram‐positive and Gram‐negative bacteria, this study addresses a critical gap by focusing on antibiotic‐resistant strains [34]. Herein, we report the novel synthesis of AgNPs decorated on mesoporous silica‐coated MWCNTs (MSCNTs), employing the extract of *Angelica gigas* Nakai processed by hot‐melt extrusion (HAE) as a green stabilizing and reducing agent (Scheme 1). We systematically compare the formation of AgNPs with and without HAE and evaluate the synergistic effects of this unique composition against resistant bacteria, specifically MRSA. The prepared MSCNT formulations were comprehensively characterized by transmission electron microscopy (TEM), energy‐dispersive spectroscopy (EDS), and X‐ray diffraction (XRD). Crucially, we also investigated the dual therapeutic potential of these nanohybrids by assessing their cytotoxicity and apoptosis‐inducing effects on both normal human keratinocyte (HaCaT) cells and triple‐negative breast cancer (MDA‐MB‐231) cells. This research aims to provide a robust foundation for developing innovative, green‐synthesized nanomaterials with enhanced dual antibacterial and anticancer properties.

## 2. Method

### 2.1. Materials

Tetraethyl orthosilicate (TEOS), cetyltrimethylammonium bromide (CTAB), N‐[3‐(trimethoxysilyl) propyl] ethylene diamine (TSD), and MWCNT (diameter 20–40 nm, length 5–15 μm) were obtained from TCI Chemicals. Silver nitrate (AgNO_3_) and crystal violet were obtained from Daejung, Korea. High glucose Dulbecco’s modified Eagle medium (DMEM), Roswell Park Memorial Institute (RPMI) medium, penicillin–streptomycin (P/S), and fetal bovine serum (FBS) were purchased from Thermo Fisher Scientific. Acridine orange (AO), ethidium bromide (EB), and propidium iodide (PI) were purchased from Sigma‐Aldrich. Mueller–Hinton agar (MHA), Mueller–Hinton broth (MHB), and D‐(+) glucose were purchased from MB Cell. The cell viability assay kit (CELLO MAX) was purchased from MediFab.

### 2.2. Preparation of HAE

Following previous studies, the HME‐processed *A. gigas* Nakai (HME‐AGN) was processed [35]. One gram of HME‐AGN was weighed and placed in distilled water. The active components of HME‐AGN were extracted by sonication in a water bath for 1 h. The HAE was filtered at room temperature, and the solvent was volatilized using a rotary evaporator. HAE was recovered to concentrate with distilled water and kept at 4°C.

### 2.3. Preparation of MSCNT

The MSCNT was manufactured by modifying the previous method [33]. For the attachment of CTAB to the MWCNT surface, MWCNTs were added to 137 mM CTAB solution and bath‐sonicated for 3 h, followed by 1 h probe tip sonication. The CTAB solution containing MWCNTs was added with 100 mL of distilled water, and 2 M NaOH was added while stirring at 60°C. TEOS solution diluted in ethanol (1:4 = TEOS:EtOH) was added with stirring and kept at 60°C for 12 h. The reactant was centrifuged at 1,851 × g for 20 min to remove the supernatant. The MSCNT was washed three times with 12.5 mM NH_4_NO_3_ ethanol solution.

### 2.4. Synthesis of MSCNT‐AgNO_3_ and MSCNT‐HAE

The synthesis of MSCNT‐AgNO_3_ and MSCNT‐HAE was performed following the methodology previously reported [34]. The process involved the preparation of a 60 mM AgNO_3_ solution, followed by the addition of TSD to attain an AgNO_3_/TSD solution with an AgNO_3_:TSD molar ratio of 1:1. Subsequently, 0.96 mL of the AgNO_3_/TSD solution was placed in 11.04 mL of ethanol, which contained 20 mg of MSCNT. After sonication in a water bath for 2 min, the mixture was allowed to stir for 3.5 h at 60°C to finally obtain MSCNT‐AgNO_3_. The mixture of AgNO_3_/TSD solution, ethanol, and MWCNTs was sonicated in a water bath for 2 min, followed by stirring at 60°C for 2 h. HAE (1, 5, or 10 mg/mL) was added to the 5 mL mixture and stirred for an additional 1.5 h to prepare MSCNT‐HAE (Table 1). After stirring, all reactants were washed three times with distilled water and dried.

**Table 1:** Composition of MSCNT formulations.

	MSCNT	AgNO_3_	HAE
MSCNT‐AgNO_3_	O	O	
MSCNT‐HAE 1	O	O	1 mg/mL
MSCNT‐HAE 2	O	O	5 mg/mL
MSCNT‐HAE 3	O	O	10 mg/mL

*Note:* MSCNT: Multiwalled carbon nanotube (MWCNT) coated with mesoporous silica; HAE: Hot‐melt extrusion (HME)‐*Angelica gigas* Nakai (AGN) extract.

### 2.5. Characterization

The morphological characterization of MSCNT formulations was conducted via TEM analysis using a JEM‐2100F microscope at an accelerating voltage of 200 kV (JEOL, Tokyo, Japan). Before analysis, samples were prepared for TEM analysis by dispersing them in ethanol and depositing them on a copper grid. Elemental analysis was performed using an EDS equipped with a TEM. The crystal structure was determined using a PANalytical X‐ray diffractometer (X’pert Pro MPD, Netherlands) by detecting XRD at 5°–80° within a 2*θ* range. The chemical compositions were studied using an FTIR spectrometer (Nicolet iN10, Thermo Fisher Scientific, USA) in the range of 400–4000 cm^−1^.

### 2.6. Minimum Inhibitory Concentration (MIC) and Minimum Bactericidal Concentration (MBC)

MRSA (ATCC 43300) cultures on MHB were incubated at 37°C overnight. The MIC was identified according to a previously established protocol [36]. Serial dilutions of the sample with MHB were injected into a test tube containing 1.5 mL of MRSA culture (OD 600 = 0.1) and incubated for 24 h at 37°C. Following the MIC analysis, 50 μL of the solution in each well was plated onto MHA plates and incubated for 24 h at 37°C. The MIC was identified as the lowest concentration at which the test tube appeared as clear as the media after incubation. The MBC was determined to be the concentration at which no colonies formed.

### 2.7. Growth Kinetics

A 96‐well plate was prepared by adding 100 μL of MRSA, diluted to an absorbance value of 0.1 at 600 nm, to each well. Subsequently, to the wells containing the bacteria suspension, 100 μL of each sample was added. At set times (0, 1, 2, 4, 8, 12, and 24 h), the microplates were observed for absorbance at 600 nm using a microplate reader. Control is the absorbance of a well containing distilled water, the sample dilution solvent.

### 2.8. Antibiofilm Activity

The antibiofilm evaluation was conducted using a modified methodology based on the research reported previously [37]. The bacterial cultures were prepared to an OD 600 of 0.1 using a medium with 0.5% glucose. Subsequently, 100 μL of each sample concentration and 100 μL of the bacterial suspension were added to a 96‐well microplate and incubated for 24 h at 37°C for the biofilm inhibition study. To assess biofilm destruction, a new 96‐well plate with 100 μL of MRSA suspension in each well was incubated for 24 h. The MRSA culture was then withdrawn, and 100 μL of the sample was processed and incubated for a further 24 h. The cultures were removed from each well, and any remaining bacterial residue was removed with PBS. The plates were dried at room temperature, and 100 μL of ethanol was injected into each well for fixation. Following the removal of ethanol and drying of the wells, 200 μL of 1% crystal violet was added to each well, and the biofilm was stained for 30 min. Each well was washed to remove any remaining crystal violet, and the plates were dried at room temperature. After the addition of 200 μL of ethanol and elution for 30 min at room temperature, 100 μL of each well was moved to a new plate and scanned using a microplate reader at 600 nm.

### 2.9. Cytotoxicity

The cytotoxicity of the samples against normal and cancer cells was investigated using the WST assay. HaCaT and MDA‐MB‐231 were cultured in DMEM and RPMI media containing 10% FBS and 1% P/S, respectively, at 5% CO_2_ and 37°C. Cells cultured in T25 flasks were seeded into 96‐well plates at 1 × 10^4^ cells/well and incubated for 24 h. Each well of the cultured 96‐well plate was treated with different concentrations (1000–15 μg/mL) of the sample. After sample treatment, the plates were incubated in an incubator for 24 h. Each well was treated with 10 μL of WST reagent, and the absorbance was measured using a plate reader (SpectraMax ABS Plus Microplate Reader) at 450 nm after 1 h. Using the measured absorbance, cell viability was calculated as shown in the following equation:

(1) Cell viability %=Sample absorbanceControl absorbance×100.

### 2.10. Fluorescent Staining

MDA‐MB‐231 cells cultured in 24‐well plates were treated with 250 and 125 μg/mL of MSCNT‐AgNO_3_ and MSCNT‐HAE_3_. After 24 h of incubation at 37°C, the media in each well were removed and washed with PBS. To determine the morphological changes of the cells in the control and treated groups, they were observed under a microscope. Under dark conditions, cells were stained by treating different wells with 10 μL of AO/EB (1:1 v/v) and PI and washed with PBS. The stained cells were observed using a fluorescent microscope (Olympus CKX53, Japan).

### 2.11. Flow Cytometry

The rate of cell death was quantified using flow cytometry. HaCaT and MDA‐MB‐231 cells treated with MSCNT‐AgNO_3_ and MSCNT‐HAE 3 (250 μg/mL) for 24 h were washed with PBS and collected for the identification of the apoptotic phase. The cells were resuspended in 1X binding buffer. Annexin V‐FITC and PI were used to stain the cells in sequence. The stained cells were filtered using FACS filter tubes and analyzed using FACS (FACSymphony A3, Becton Dickinson).

### 2.12. Statistical Analysis

The data were analyzed using one‐way analysis of variance (ANOVA) for comparison. All experimental analyses were performed in triplicate. Duncan’s multiple range test (DMRT) was used to analyze the results statistically (*p* < 0.05). SAS software (SAS Institute Inc., USA) was used for all data.

## 3. Results and Discussion

### 3.1. TEM Analysis

In the MSCNT‐AgNO_3_ formulation, ethanol was utilized as a reducing agent. Conversely, in the MSCNT‐HAE formulation, HAE was added as an additional reducing and stabilizing agent alongside ethanol. The formation of AgNPs on the MSCNT was confirmed by TEM images (Figure 1(a)). The NPs formed were found to be mostly spherical. In formulations containing the HME‐AGN extract, MSCNT‐HAE 2 and MSCNT‐HAE 3 were confirmed to have AgNPs aggregated on the MSCNT surface. The presence of Ag ions was confirmed through EDS mapping and spectrum (Figures 1(b) and 1(c)). MSCNT‐AgNO_3_ and MSCNT‐HAE 3 were confirmed to have formed AgNPs in the pores of mesoporous silica. In MSCNT‐HAE 3, AgNPs were confirmed to be uniformly distributed in the MWNCT. Mesoporous silica morphology leads to the formation of uniform AgNPs [38]. In our previous research, we demonstrated the formation of mesoporous silica with a pore size of approximately 4.409 μm on CNTs [34]. The formation of AgNPs can be enhanced by the addition of plant extracts, and we confirmed the formation of homogeneous particles at the highest concentration. The uniform size of AgNPs may improve their antimicrobial activity by enabling the sustained release of silver ions [39].

Figure 1(a) Transmission electron microscopy (TEM) images, (b) EDS mapping (red points: Ag), and (c) EDS spectrum of (A) MSCNT‐AgNO_3_, (B) MSCNT‐HAE 1, (C) MSCNT‐HAE 2, and (D) MSCNT‐HAE 3.

**Figure:**
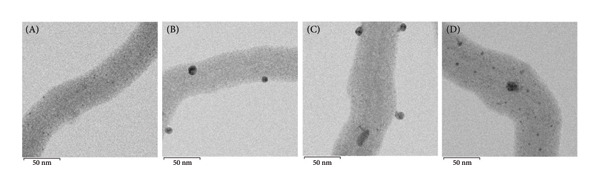
(a)

**Figure:**
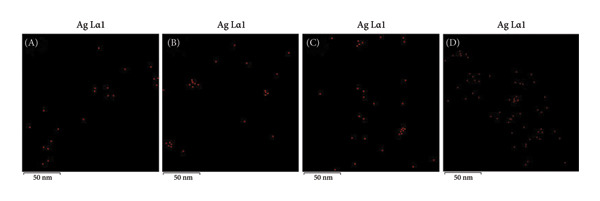
(b)

**Figure:**
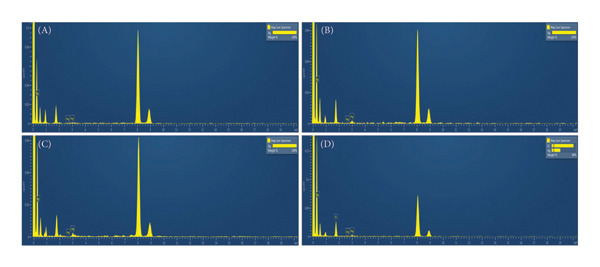
(c)

### 3.2. XRD Analysis

**Figure 2:**
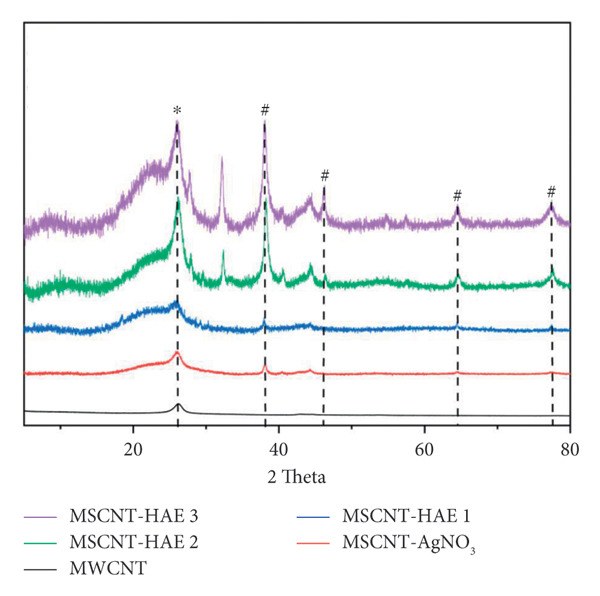
XRD patterns of MWCNT and MSCNT formulations. ^∗^Carbon; ^#^Ag.

XRD was used to study the structure and chemical composition of the MSCNT formulation (Figure 2). The diffraction peak that appears at approximately 26° corresponds to carbon atoms (JCPDS No. 01–0646) [38] and was identified in all MSCNT formulations. The diffraction peaks appearing at approximately 38, 46, 64, and 78° correspond to Ag (111), (200), (220), and (311) planes (JCPDS No. 04‐0783), respectively [40]. The peak corresponding to Ag was observed to be low in MSCNT‐AgNO_3_ and MSCNT‐HAE 1, and the intensity of this peak increased with higher concentrations of HAE. The XRD pattern further confirmed the formation of AgNPs on MSCNTs [26]. Additionally, the peak that forms at approximately 33° corresponds to a face‐centered cubic crystal structure of silver oxide (Ag_2_O) (01‐076‐1393) [41, 42]. This may be attributed to the partial oxidation of silver, likely caused by the changes in reduction conditions at higher concentrations of HAE. TEM and XRD results demonstrated that the addition of plant extract could further enhance particle formation. HAE can be used as a reducing agent for the formation of AgNPs, with increasing concentration indicating a higher degree of reduction.

### 3.3. FTIR Analysis

**Figure 3:**
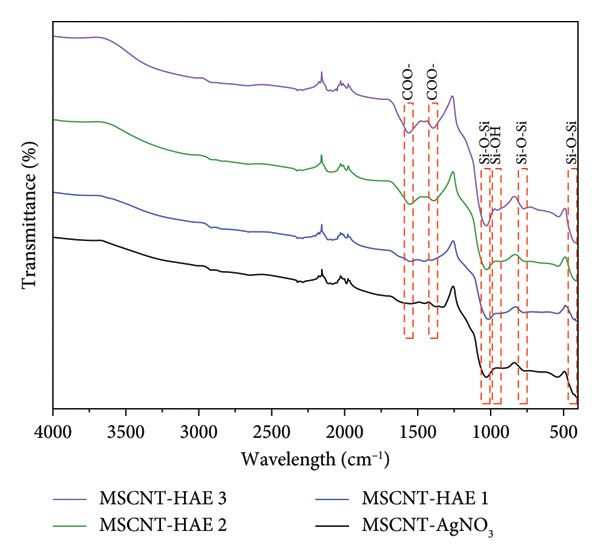
FTIR spectrum of MSCNT formulations.

FTIR was performed to confirm the changes in the functional groups (Figure 3). The peaks observed at 437, 776, and 1032 cm^−1^ in all samples are attributed to the elastic stretching of Si–O–Si bonds. Specifically, the peak at 437 cm^−1^ corresponds to Si–O–Si strain vibrations, while the peaks at 776 and 1032 cm^−1^ are associated with symmetric and asymmetric Si–O–Si vibrations, respectively [43]. Additionally, the peak at 952 cm^−1^ is linked to Si–OH vibrations [44]. The intensity of the peaks around 1390 and 1560 cm^−1^ increases with higher concentrations of the added extract. These peaks correspond to the symmetric and asymmetric stretching vibrations of carboxylate (COO^−^), respectively [45]. The increased intensity of these peaks may indicate a higher concentration of active compounds, such as phenols and flavonoids, in the extract. Our previous research has shown that the total phenol content of MSCNTs rises proportionally with the concentration of the added extract [34].

### 3.4. In Vitro Antibacterial Activities

MRSA represents one of the most prevalent MDR strains, posing significant challenges for treatment with conventional antibiotics [40]. Ampicillin, used as a control in MRSA, did not inhibit bacterial growth even at the highest concentration of 1 mg/mL (Figure 4(a)). MSCNT‐AgNO_3_, MSCNT‐HAE 1, and MSCNT‐HAE 2 all became as transparent as bacteria‐free media up to 0.25 mg/mL (Figures 4(b), 4(c), and 4(d)). For MSCNT‐HAE 3, the lowest concentration of 0.125 mg/mL was confirmed as the MIC (Figure 4(e)). The MIC of each sample was determined for MRSA, and the growth curve was monitored for up to 24 h (Figure 4(f)). All formulations of MBC were found to be 0.5 mg/mL (Table 2). The control group treated with D.W for 24 h showed bacterial growth with an OD 600 value below approximately 0.4, and all MSCNT formulations showed OD 600 values below 0.1. All samples exhibited sustained inhibition of MRSA growth for up to 24 h. Among them, MSCNT‐HAE 3 demonstrated the most pronounced growth‐suppressive effect, which is likely attributable to the higher density of AgNPs deposited on the MSCNT surface. CNTs demonstrate antibacterial properties by mechanisms including microbial penetration, leading to cell membrane disruption and production of ROS [46]. AgNPs synthesized from mesoporous structures display enhanced antibacterial efficacy attributed to the sustained release of silver and the capacity to encapsulate a substantial quantity of AgNPs per volume unit [47]. Consequently, the integration of MSCNTs with mesoporous silica coatings and AgNPs demonstrates potential antibacterial activity against MRSA under in vitro conditions.

Figure 4In vitro antibacterial activity of (a) ampicillin, (b) MSCNT‐AgNO_3_, (c) MSCNT‐HAE 1, (d) MSCNT‐HAE 2, and (e) MSCNT‐HAE 3 against MRSA. (f) Growth curve of MSCNT formulations. Data are expressed as mean ± standard deviation (*n* = 3).

**Figure:**
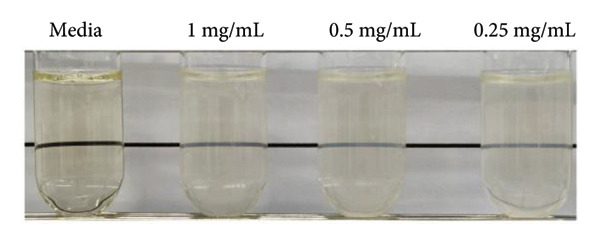
(a)

**Figure:**
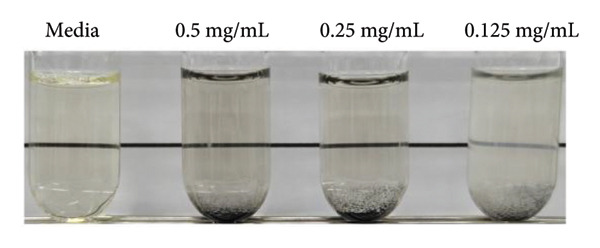
(b)

**Figure:**
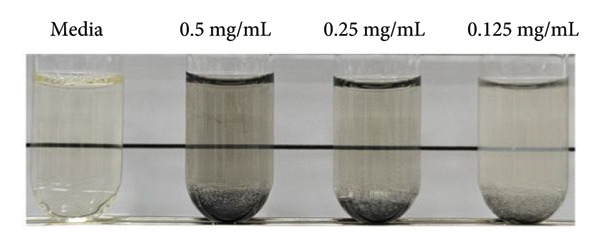
(c)

**Figure:**
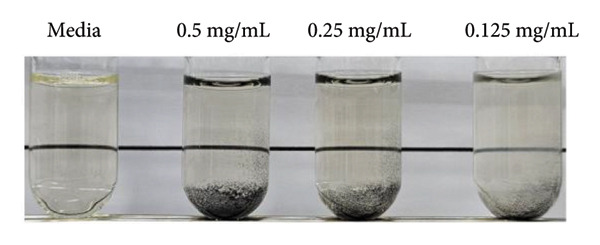
(d)

**Figure:**
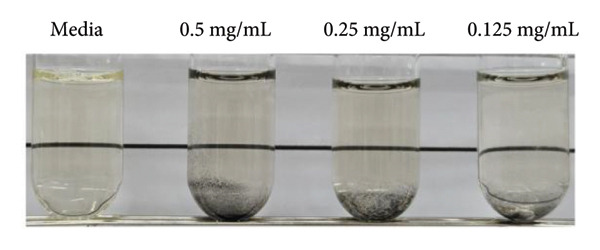
(e)

**Figure:**
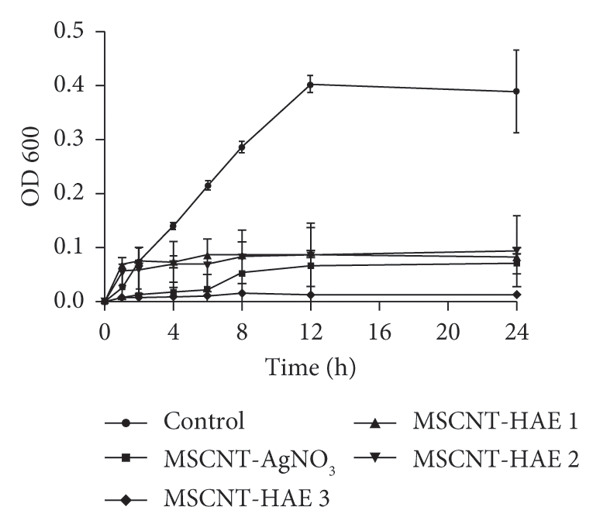
(f)

**Table 2:** MIC and MBC values of MSCNT formulations against MRSA.

Sample	MIC (μg/mL)	MBC (μg/mL)
MSCNT‐AgNO_3_	250	500
MSCNT‐HAE 1	250	500
MSCNT‐HAE 2	250	500
MSCNT‐HAE 3	125	500

*Note:* Data are expressed as mean values (*n* = 3).

### 3.5. Antibiofilm Activity

MSCNT formulations of 125, 250, 500, and 1000 μg/mL were used to evaluate biofilm formation inhibition and destruction of MRSA (Figure 5). AgNPs can inhibit biofilm formation against *Escherichia coli*, *Pseudomonas aeruginosa*, and *S. aureus* [48, 49]. The antibiofilm activity of AgNPs has also been reported against antibiotic‐resistant bacterial strains [50, 51]. The surface functionalization of AgNPs with plant‐derived compounds enhances their stability and improves antibacterial performance. MSCNT‐AgNO_3_ showed improved inhibitory ability in a concentration‐dependent manner but showed low activity at low concentrations, such as 125 and 250 μg/mL. Among the MSCNT‐HAE formulations, all except MSCNT‐HAE 2 showed high activity. MSCNT‐HAE 3 showed consistently high inhibition activity starting from low concentrations.

Figure 5(a) Inhibition and (b) destruction effect of the biofilm with MSCNT formulations. Data are expressed as mean ± standard deviation (*n* = 3). Different letters (A–E) indicate the statistically significant differences between groups (*p* < 0.05).

**Figure:**
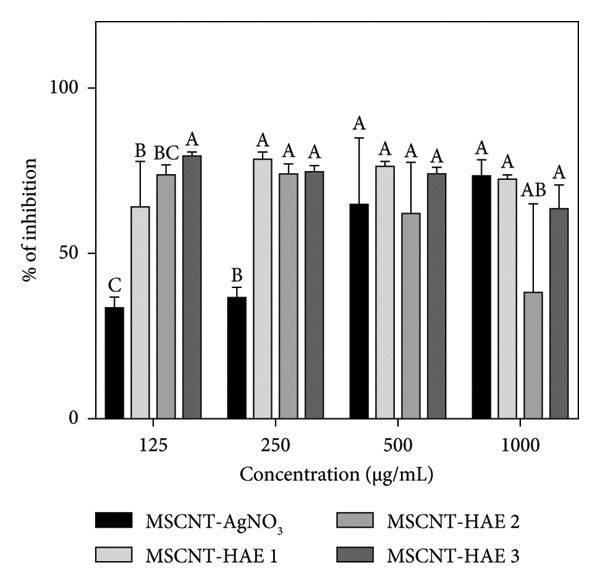
(a)

**Figure:**
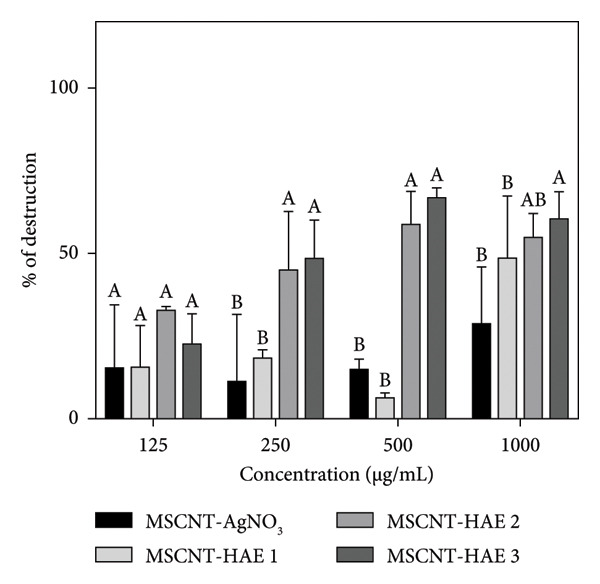
(b)

While preventing biofilm formation is essential, the disruption of mature biofilms is particularly critical for effective antimicrobial strategies. Mature biofilms cause serious problems as they are resistant to conventional antimicrobials [52]. At a concentration of 125 μg/mL, there was no significant difference between the MSCNT‐AgNO_3_ and MSCNT‐HAE formulations. At higher concentrations, treatment of the MSCNT‐HAE formulation had a significant effect on biofilm removal, with MSCNT‐HAE 3 showing particularly high activity. MSCNT‐HAE 3 exhibited the most significant inhibitory and destructive activities, and its capacity to inhibit bacterial growth and prevent biofilm formation was also demonstrated.

### 3.6. Cell Viability and Cell Staining

To assess the safety profile of the MSCNT formulations at concentrations ranging from 15 to 1000 μg/mL, HaCaT cells, immortalized human keratinocytes, were used as a representative normal cell line. In HaCaT cells, viability decreased after 24 h of treatment, particularly at higher concentrations (Figure 6(a)). Among the formulations, MSCNT‐HAE 3 showed higher cell viability compared to the others, starting at 62 μg/mL. The IC_50_ values for HaCaT cells were 423.43 μg/mL for MSCNT‐AgNO_3_, 297.21 μg/mL for MSCNT‐HAE 1, 416.40 μg/mL for MSCNT‐HAE 2, and 759.07 μg/mL for MSCNT‐HAE 3, making MSCNT‐HAE 3 the least toxic (Table 3). Additionally, the MIC for the MRSA of MSCNT‐HAE 3 demonstrated no cytotoxicity in HaCaT cells. CNTs have been investigated for breast cancer therapy, with their cytotoxic effects potentially enhanced through functionalization [53]. Cytotoxicity against MDA‐MB‐231 cells was assessed at the same concentrations used for HaCaT cells, and MSCNT‐HAE 3 demonstrated greater cytotoxic effects on MDA‐MB‐231 cells compared to HaCaT cells (Figure 6(b)). The IC_50_ values were 431.75 μg/mL for MSCNT‐AgNO_3_, 349.73 μg/mL for MSCNT‐HAE 1, 289.88 μg/mL for MSCNT‐HAE 2, and 153.97 μg/mL for MSCNT‐HAE 3 (Table 3). The concentration of AGN extract added may influence the differential cytotoxic response of MSCNT‐HAE 3 in normal and cancer cells. AgNP‐MWCNT nanocomposites, synthesized using plant extracts, have been shown to inhibit cancer cells via a green synthesis method using plant‐based reducing agents [30]. MDA‐MB‐231 cells were exposed to 250 μg/mL and 125 μg/mL of MSCNT‐AgNO_3_ and MSCNT‐HAE 3, respectively, for 24 h, followed by observation for morphological changes and fluorescent staining using AO/EB and PI (Figure 6(c)). In the control group, cells exhibited uniform distribution under microscopic observation and AO/EB staining. After treatment with MWCNT‐AgNO_3_ and MSCNT‐HAE 3, the number of viable cells decreased, and fluorescent green‐stained nuclei were observed, indicating early cell death through chromatin condensation or fragmentation [54]. PI staining further identified nuclear damage caused by the treatments. Compared to the control group, the number of PI‐stained cells increased, although at lower concentrations, the proportion of stained cells was reduced. These findings suggest that MSCNT‐HAE 3 can serve as a therapeutic agent capable of inhibiting cancer cell growth while exhibiting low cytotoxicity in normal cells. The AgNPs synthesized from the extract demonstrated a higher activity against tumor cells and lower toxicity in nontumor cells [55].

Figure 6Cell viability of (a) HaCaT and (b) MDA‐MB‐231 cells by WST assay and (c) MDA‐MB‐231 cell staining with AO/EB and PI (scale bar: 200 μm). Different letters (A–E) indicate the statistically significant differences between groups (*p* < 0.05).

**Figure:**
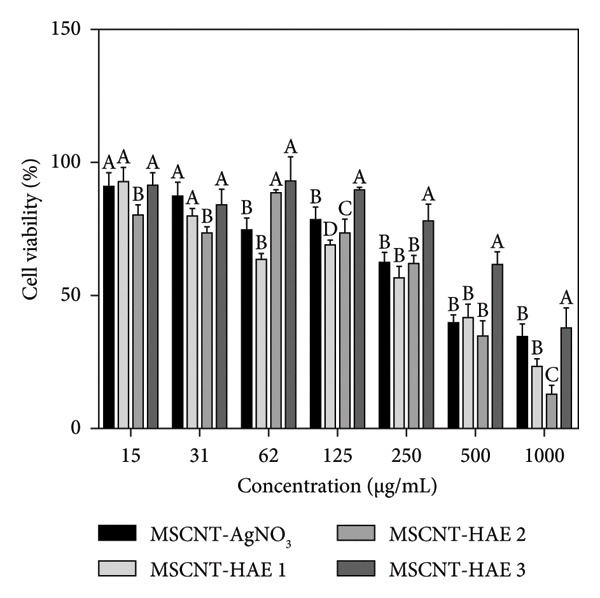
(a)

**Figure:**
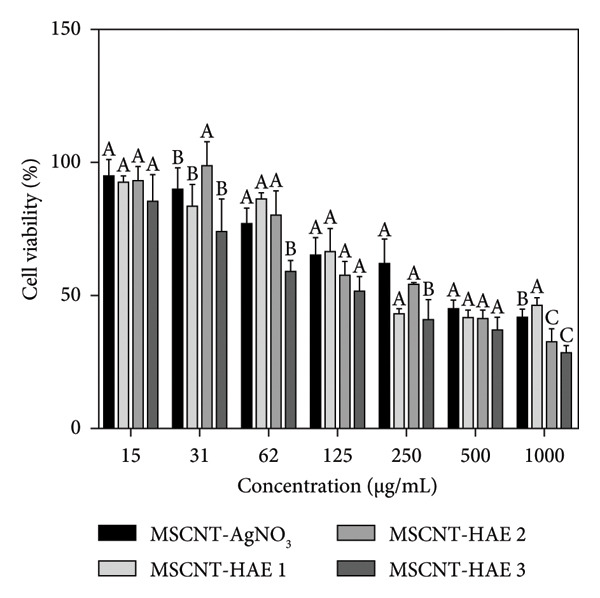
(b)

**Figure:**
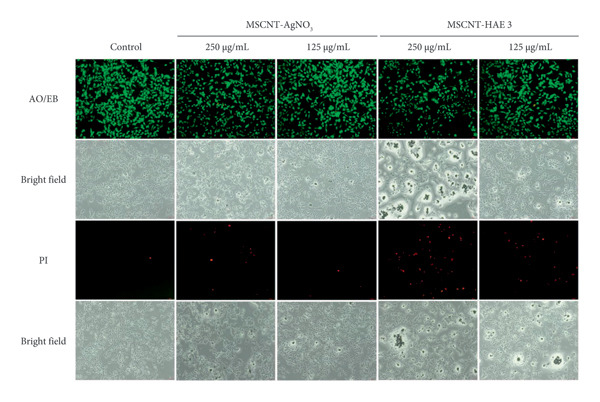
(c)

**Table 3:** IC_50_ (μg/mL) of MSCNT formulations in HaCaT and MDA‐MB 231.

Samples	HaCaT	MDA‐MB‐231
	IC_50_ (μg/mL)	
MSCNT‐AgNO_3_	423.43 ± 60.45	431.75 ± 119.06
MSCNT‐HAE 1	297.21 ± 73.61	349.73 ± 67.13
MSCNT‐HAE 2	416.40 ± 40.43	289.88 ± 34.95
MSCNT‐HAE 3	759.07 ± 118.11	153.97 ± 26.53

*Note:* Data were expressed as mean ± standard deviation (*n* = 3).

### 3.7. Apoptosis Analysis of MSCNT‐AgNO_3_ and MSCNT‐HAE3

One of the objectives of this study was to examine the correlation between reduced cell viability and apoptosis in MDA‐MB‐231 cells following treatment with MSCNT formulations. MDA‐MB‐231 cells treated with MSCNT‐AgNO_3_ and MSCNT‐HAE 3 were stained with Annexin V and PI to assess apoptosis (Figures 7(a) and 7(b)). In the control group, the percentages of apoptotic and necrotic cells were 1.17% and 2.03%, respectively. Treatment with MSCNT‐AgNO_3_ and MSCNT‐HAE 3 resulted in a significant increase in Annexin V staining, indicating a rise in both early and late apoptotic cells. The percentage of apoptotic cells was 14.47% for MSCNT‐AgNO_3_ and 25.3% for MSCNT‐HAE 3, with a greater increase in late apoptotic cells compared to early apoptotic ones (*p* < 0.05). Despite a slight increase in necrosis, MSCNT‐HAE 3 exhibited a markedly higher apoptotic rate compared to MSCNT‐AgNO_3_, resulting in an improved apoptosis/necrosis ratio of 2.25 versus 1.63, indicating the potential for selective induction of apoptosis over nonspecific cytotoxicity (Figure 7(b)). The morphology of mesoporous silica has been shown to enhance cytotoxicity and apoptosis by improving the delivery of anticancer drugs [56]. MSCNT‐HAE 3 treatment induced more cell death in MDA‐MB‐231 cells than MSCNT‐AgNO_3_, demonstrating the most pronounced apoptotic effect among the tested formulations. The apoptosis of HaCaT cells, a normal cell, was also investigated by Annexin V and PI staining (Figures 7(c) and 7(d)). In the control group, 98.9% of normal cells were stained, while 97.3% of MSCNT‐AgNO_3_ and 98.9% of MSCNT‐HAE 3 were stained, confirming that neither formulation induced cell death in normal cells. Consequently, it was determined that neither formulation induced apoptosis in normal cells. This induction of apoptosis was specific to cancer cells.

Figure 7Flow cytometry analysis of MDA‐MB‐231 cells and HaCaT cells treated with MSCNT‐AgNO_3_ and MSCNT‐HAE 3. Quadrants showing Annexin V/PI fluorescence are numbered as follows: apoptosis (Q2 [late apoptosis], Q4 [early apoptosis]), Q1 (necrosis), Q3 (normal). Data were expressed as mean ± standard deviation (*n* = 3). Statistical comparison between MSCNT‐AgNO_3_ and MSCNT‐HAE 3 was performed using an unpaired *t*‐test (*p* < 0.05).

**Figure:**
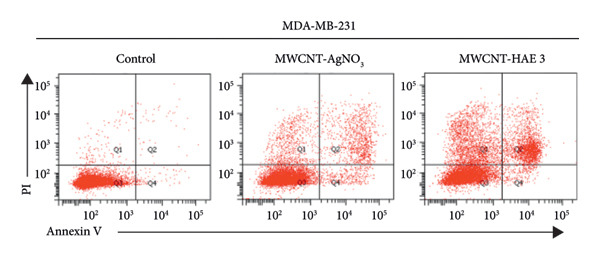
(a)

**Figure:**
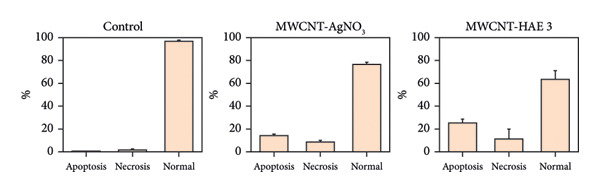
(b)

**Figure:**
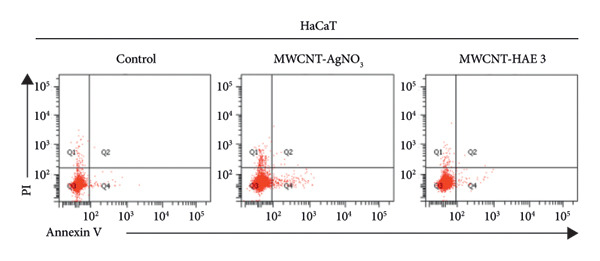
(c)

**Figure:**
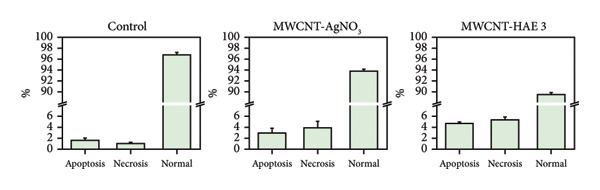
(d)

## 4. Conclusion

MSCNTs containing AgNPs have been proposed as an alternative to antibiotic resistance. MSCNTs are a carrier capable of regularly loading and delivering AgNPs. The formation of AgNPs was increased by the addition of HAE, which was confirmed by TEM and XRD analysis. Against MRSA, all formulations showed an improved antibacterial activity over antibiotics, with MSCNT‐HAE 3 suggested as the most potent antibacterial agent. Furthermore, the ability to inhibit and destroy the biofilm of MRSA was confirmed in all formulations containing AgNPs. The results of cell studies demonstrated that MSCNT formulations exhibited cytocompatibility in HaCaT cells, with MSCNT‐HAE 3 exhibiting the highest IC_50_ value. The MSCNT‐HAE 3 exhibited a greater cytotoxic effect and increased cell death in MDA‐MB‐231 cells in comparison with the MSCNT‐AgNO_3_. MSCNT formulations can be proposed as an alternative for the treatment of antibiotic‐resistant bacteria and cancer cells.

## Conflicts of Interest

The authors declare no conflicts of interest.

## Author Contributions

Su‐Ji Ryu: conceptualization, methodology, investigation, and writing–original draft preparation. Han‐Sol You: investigation and data curation. Hye‐Min Kim: investigation. Hyo‐Jin An: writing–review and editing and project administration. Jong‐Suep Baek: funding acquisition, writing–review and editing, supervision, and project administration.

## Funding

This research was supported by a grant of the Korea Health Technology R&D Project through the National Research Foundation (NRF) and the Korea Health Industry Development Institute (KHIDI), funded by the Ministry of Science and ICT, Republic of Korea, and the Ministry of Health & Welfare, Republic of Korea (Grant Number: RS‐2023‐00262645).

## Data Availability

Data are available from the corresponding author upon reasonable request.
